# The impact of polymerase chain reaction (PCR) on diagnosis and management of infectious uveitis at a tertiary care facility

**DOI:** 10.1186/s12348-021-00276-w

**Published:** 2022-01-06

**Authors:** Julia Fallon, Swati Narayan, Jun Lin, Jodi Sassoon, Stephanie Llop

**Affiliations:** 1 Department of Ophthalmology, New York Eye and Ear Infirmary of Mount Sinai, New York, New York USA; 2 Department of Pathology, New York Eye and Ear Infirmary of Mount Sinai, New York, New York USA; 3 Department of Ophthalmology, Bascom Palmer Eye Institute, Miami, Florida USA

**Keywords:** Infectious uveitis, Polymerase chain reaction (PCR), Diagnostic utility

## Abstract

**Background:**

Polymerase Chain Reaction (PCR) is a well-accepted adjunct in the management of infectious uveitis. In turn, few reports in the literature have evaluated how PCR then impacts patient care. This study aims to evaluate the impact of PCR sampling on diagnosis and treatment of infectious uveitidies at a large tertiary care facility.

**Main body:**

This is a retrospective, observational study of patients with aqueous and vitreous PCR samples obtained from 2014 to 2019. The study was undertaken at a single institution. At least one follow up visit following results of PCR testing was required for inclusion. If a patient had multiple PCR samples taken, only the first sample was included. The patients were divided into three categories based on pre-sampling diagnosis. A chi-square test was used to analyze the data. 108 cases were available for analysis. PCR did not change diagnosis or management in any of the cases where pre-sampling diagnosis carried a high clinical suspicion for negative PCR. Overall, the results of PCR testing had a more significant impact on diagnosis in those cases where pre-sampling diagnosis was unknown versus those where it was confirmatory in nature, thus presumed to be related to an infectious entity tested by PCR (74% vs. 29%, *p* = 0.00006). The rate of treatment change based on PCR was similar between those cases where there was a high clinical suspicion for positive PCR and those where pre-sampling diagnosis was unknown (32% vs. 33%, *p* = 0.95). Further analyzing specimens separately depending on source of sample, this pattern persisted for aqueous samples, with PCR showing a more significant impact on diagnosis in those cases where the diagnosis was unknown versus those where sampling was confirmatory (86% vs. 31%, *p* = 0.00004). The rate of change in treatment between the two groups was similar (35% vs. 31%, *p* = 0.79). Vitreous samples followed a similar pattern with a higher rate of diagnosis change for those cases where pre-sampling diagnosis was unknown and a similar rate in treatment change between the two groups, however this did not reach statistical signifigance (44% vs. 25%, *p* = 0.28; 27% vs. 33%, *p* = 0.74).

**Conclusion:**

There is no well-defined algorithm as to when to employ PCR testing in uveitis. As expected, in our experience, it has the largest impact on diagnosis when the diagnosis is unknown, however even when confirmatory in nature, it continues to impact patient management.

## Background

Uveitis describes a range of diseases marked by inflammation within the eye, which can occur secondary to various causes, including autoimmune disease, infectious agents and cancer [1–5]. The diagnosis of uveitis carries increased risk of ocular complications including glaucoma, cataract and blindness [6]. Recent epidemiologic studies report the prevalence of uveitis at 5.4 per 1000 residents in the United States [1], with noninfectious uveitis accounting for majority of the cases [2], whereas infectious uveitis is more commonly found in the developing world [3]. Geography further dictates prevalence, for example herpetic uveitis is the most common infectious etiology in Saudi Arabia, whereas tuberculosis is the most common in India [3]. Even with many known infectious and noninfectious etiologies of uveitis, it is estimated anywhere from 30 to 60% cases of uveitis are idiopathic [1, 3, 7]. Classification is based on anatomical location, with anterior uveitis accounting for most cases [4, 7, 8]. Diagnostic workup is tailored to each case, as laboratory tests and studies ordered vary depending on clinical presentation, the provider, geography and resources of the health care system [9, 10].

Identifying infectious uveitis and beginning adequate antimicrobial therapy early in disease course is essential to prevent ocular complications. Toxoplasmosis and Herpes viruses account for the most commonly seen infectious uveitides in the United States [11–13]. Polymerase Chain Reaction (PCR) has emerged as a useful diagnostic tool for infectious uveitis, showing high sensitivity and rapid results, in comparison to gold standards like culture or fundus examination [14–17]. There are two main types of PCR used: multiplex and real-time. Multiplex PCR, also known as qualitative PCR, helps to determine if an agent is present within a specimen, without commenting on its quantity. A recent literature review has shown that it can be successful at differentiating between two to five causative agents, thus limiting the need for biopsy which carries higher morbidity [18]. Real-time PCR, also known as quantitative, can be used to determine the level of disease burden. By quantifying the amount present, real-time PCR helps determine if the pathogen is likely a contaminant or active in the disease process. When used together, these two types of PCR can help to identify and confirm a causative infectious agent [19]. PCR is now widely accepted as a useful adjunct to help diagnose cases of infectious uveitis; however, few centers have since published their experience with PCR and its effect on management in the literature [20–25]. The present study was undertaken to report the experience of PCR testing in various clinical scenarios at a large tertiary care center in New York City.

## Methods

Records were reviewed of all Department of Ophthalmology patients with at least one PCR study performed seen at New York Eye and Ear Infirmary of Mount Sinai, a tertiary care center located in New York City, between 2014 and 2019. The study was conducted in accordance with the IRB and HIPAA regulations. Patients were deemed appropriate for inclusion if they had a sample of ocular fluid (aqueous or vitreous) sent for PCR testing. Aqueous fluid was removed from the eye via an anterior chamber paracentesis using a 30 G needle and 1 mL syringe. Vitreous fluid was removed via vitreous tap using a 25 G needle and 3 mL syringe, or via pars plana vitrectomy. Samples were sent to ARUP Laboratories (Salt Lake City, Utah) for PCR analysis. Multiplex (qualitative) PCR was run on samples for detection of Herpes Simplex Virus (HSV), Varicella Zoster Virus (VZV), Cytomegalovirus (CMV), *Toxoplasmosis gondii* (Toxoplasmosis) and *Mycobacterium tuberculosis* (M. tb). Real-time (quantitative) PCR was used for detection of Epstein-Barr Virus (EBV). To be included in the study, patients required at least one follow up visit in clinic following results of PCR testing. Patients with primary corneal pathologies such as interstitial keratitis were excluded from the study.

Patient charts were accessed and reviewed through Meditech (Westwood, Massachusetts)**.** Clinical information was collected pre- and post- PCR sampling, including visual acuity and anatomical classification of uveitis. The uveitis was defined in accordance with the SUN Working Group nomenclature [4]. Clinical diagnosis and treatment before and after the results of PCR testing resulted were recorded. The cases were then divided into three groups based on pre-testing diagnosis: “High clinical suspicion for a negative PCR result”, “High clinical suspicion for an infectious uveitis due to HSV, VZV, CMV, Toxoplasmosis or M. tb.”, and “Unknown etiology, possibly secondary to an infectious agent tested for by PCR”. Treatment data prior to and following PCR testing was collected. Initial treatment regimens were grouped into categories including immunosuppression (including by mouth (PO), injections or intravitreal), antiviral treatments (including PO, intravitreal or intravenous), antibiotic treatments (including PO, intravitreal or intravenous), pars plana vitrectomy and lastly any topical modality including steroids, cycloplegics or intraocular pressure lowering drops. A change in management was defined as addition, subtraction or change in dosage of any treatment agent secondary to a positive or negative PCR result. A Chi-squared test was used to analyze the data.

## Results

137 cases were eligible for inclusion in this study. 21 cases were excluded, 20 of which did not meet our criteria for follow-up, at least one clinic visit following results of PCR testing, and 1 case that had an invalid toxoplasmosis PCR result. 116 cases with PCR studies were available for analysis. Only the first PCR sample sent from the patient was included leaving the final sample size with 108 cases. Baseline clinical and demographic data is summarized in Table 1.

**Table 1:** Patient Demographics and Pre-Sampling Data

Age	
	51.2 (mean)
	10–89 (range)
Gender	
Male	59 (55%)
Female	49 (45%)
Site of PCR	
Aqueous Tap	65 (60.2%)
Vitreous Tap	43 (39.8%)
Presenting Visual Acuity (logMAR)	
Range	0.00–3
Median	1.2
According to anatomical site	
Anterior uveitis	28 (25.9%)
Intermediate uveitis	2 (2%)
Posterior uveitis	16 (14.8%)
Panuveitis	45 (41.7%)
Anterior + intermediate uveitis	4 (3.7%)
According to duration	
Acute uveitis	48 (44.4%)
Chronic uveitis	55 (51%)
Recurrent uveitis	5 (4.6%)
Pre-sampling diagnosis	
Pre-sampling diagnosis not related to PCR	19 (17.6%)
Pre-sampling diagnosis related to PCR	28 (26%)
Unknown	61 (56%)
Initial Treatment	
Immunosupression	22 (20%)
Antivirals	45 (41.7%)
Antibiotics and/or Antifungals	30 (27.8%)
Vitrectomy	14 (13%)
Topical agents	69 (63.9%)

Mean age of participants was 51.2 years, with a median age of 51.5. 55% of study participants were male. Presenting visual acuity ranged from 20/20 (logMAR 0.00) to no light perception (logMAR 3), with median visual acuity of 20/360 (logMAR 1.2).

The majority of the samples were aqueous specimens (60.2% aqueous vs. 39.8% vitreous). The number of anterior, anterior and intermediate, panuveitis, intermediate, and posterior uveitis were 28 of the 108 (25.9%), 4 of 108 (3.7%), 45 of 108 (41.7%), 2 of 108 (2%), 16 of 108 (14.8%), respectively. The remaining cases were as follows: 7 of presumed bacterial endophthalmitis, 5 retinal detachments (primary pathology, without associated panuveitis) and 1 phacolytic glaucoma. 44.4% (48/108) were deemed acute cases, 4.6% (5/108) were recurrent and 51% (55/108) were chronic.

Cases were classified depending on initial diagnosis. There were 19 cases (17.6%) where there was high clinical suspicion for a diagnosis that was not HSV, VZV, CMV, Toxo, M. Tb related, examples of these include cases of presumed bacterial endophthalmitis or retinal detachments without underlying panuveitis. There were 28 cases (26%) where there was a high clinical suspicion for an etiology secondary to HSV, VZV, CMV, Toxoplasmosis or M. Tb. including 4 presumed cases of Toxoplasmosis, 2 cases of Progressive Outer Retinal Necrosis, 7 cases of Acute Retinal Necrosis and 2 cases of CMV retinitis. The remaining 61 cases (56%) were cases where the diagnosis was mostly unknown, but a uveitic process secondary to HSV, VZV, CMV, Toxoplasmosis or M. Tb was on the differential. Examples of unknown cases include recurrent anterior uveitis where a viral etiology was possible, cases that were acutely worsening for an unknown reason, and undifferentiated panuveitis where an infectious etiology was possible and needed to be ruled out before beginning immunosuppression. Many patients obtained multiple of these treatments simultaneously, with the most common modality being topical agents used in 63.9% of the cases. Immunosuppression, antibiotics, antivirals and vitrectomy were used in 20%, 27.8%, 41.7%, and 13% of the cases respectively.

HSV and VZV were the most common infectious agents tested for, both being run on 103 of the samples, followed by CMV on 98 of the samples, Toxoplasmosis on 59 of the samples, EBV on 27 of the samples and lastly M. tb. on 13 of the samples. The most common panel of tests was CMV, HSV, Toxoplasmosis and VZV, representing 36 of the 108 cases (33%). 25 of the 108 samples yielded positive PCR results. 13 of the 25 positive samples were from aqueous specimens (52%) and 12 of the 25 positive samples were from vitreous specimens (48%). Of samples with positive results, 7 were positive for CMV (one of these also positive for HSV), 9 were positive for HSV (one of these positive for CMV as noted previously), 6 were positive for VZV (one of these also positive for EBV), and 4 were positive for Toxoplasmosis (one of these also positive for EBV).

Table 2 summarizes treatment and diagnosis changes based on PCR results according to pre-testing diagnosis category.

**Table 2:** Diagnosis and Treatment Changes Based on PCR: All samples

Pre-sampling Groups	Total number	Diagnosis changed based on PCR (*n*)	Treatment changed based on PCR (*n*)
High clinical suspicion for negative PCR results	19	0	0
High clinical suspicion for an etiology secondary to HSV, VZV, CMV, Toxoplasmosis or M. tb.	28	8	9
Unknown etiology, possibly secondary to an infectious agent tested for by PCR	61	45	20

Overall, 53 cases (49%) had diagnosis change and 29 cases (27%) had treatment change based on results of PCR. Of note, EBV positivity or negativity had no impact on diagnosis or treatment in any of these cases. For the group where pre-testing diagnosis was not related to an infectious agent sampled with PCR, no cases had a diagnosis or treatment change based on the results of the PCR. For the group where pre-testing diagnosis was suspicious for an etiology related to PCR, 8 cases (29%) had a change in diagnosis related to PCR and 9 cases (32%) had a change in management related to PCR result. In the group where diagnosis before sampling was unknown, 45 cases (74%) had a change in diagnosis based on PCR results and 20 cases (33%) had a change in management.

The results of PCR testing had a more significant impact on diagnosis in those cases where pre-sampling diagnosis was unknown versus those where it was confirmatory in nature, thus presumed to be related to an infectious entity tested by PCR (74% vs. 29%; *p* = 0.00006). There were no cases where both pre-testing diagnosis was not suspicious for infectious agent tested by PCR and there was a change in diagnosis related to PCR results. Treatment changes based on PCR results were made at a similar rate in cases where the diagnosis was unknown compared to when a pretesting diagnosis was presumed an infectious entity tested for by PCR (33% vs. 32%, *p* = 0.95). Similar to diagnosis changes, there were no cases where treatment was changed in relation to PCR when the pre-sampling diagnosis was not suspicious for HSV, VZV, CMV, Toxoplasmosis, M. Tb.

Tables 3 and 4 further divide the cases by the type of specimen.

**Table 3:** Diagnosis and Treatment Changes Based on PCR: Aqueous

Pre-sampling Groups	Total number	Diagnosis changed based on PCR (*n*)	Treatment changed based on PCR (*n*)
High clinical suspicion for negative PCR results	6	0	0
High clinical suspicion for an etiology secondary to HSV, VZV, CMV, Toxoplasmosis or M. tb.	16	5	5
Unknown etiology, possibly secondary to an infectious agent tested for by PCR	43	37	15

**Table 4:** Diagnosis and Treatment Changes Based on PCR: Vitreous

Pre-sampling Groups	Total number	Diagnosis changed based on PCR (*n*)	Treatment changed based on PCR (*n*)
High clinical suspicion for negative PCR results	13	0	0
High clinical suspicion for an etiology secondary to HSV, VZV, CMV, Toxoplasmosis or M. tb.	12	3	4
Unknown etiology, possibly secondary to an infectious agent tested for by PCR	18	8	5

When analyzing aqueous specimens, this effect persisted with the results of PCR testing having a more significant impact on diagnosis in those cases where pre-sampling diagnosis was unknown versus those where it was confirmatory in nature, thus presumed to be related to an infectious entity tested by PCR (86% vs. 31%, p = 0.00004). The effect of PCR on management was similar between the two groups (35% vs. 31%, *p* = 0.79). For vitreous samples, the rate of change in diagnosis based on PCR was higher in those cases where the pre-sampling diagnosis was unknown versus those where it was confirmatory in nature, but this result did not reach statistical significance (44% vs. 25%, *p* = 0.28). In this subset, the rate of treatment change on PCR testing was similar between the two groups and actually slightly higher in those that underwent PCR testing for confirmation of diagnosis, but this did not reach statistical significance (27% vs. 33%, *p* = 0.74).

## Discussion

PCR has proven to be an asset to the diagnosis of infectious uveitides, however only a few centers have since published their practice patterns with PCR [20–25]. De Santos et al. recently published a similar study, although of a smaller number of cases (*n* = 28), which showed a change in treatment of around 50% of cases due to the results of PCR. This study is most representative of our “Unknown” cases subset as they evaluated PCR in situations where the clinical course was atypical or aggressive with an imminent threat to vision [20]. In contrast, two other studies analyzed clinical subsets where the utility of PCR was more confirmatory in nature [21, 22]. From India, Kharel et al. published results of PCR looking at cases of suspected infectious uveitis [21]. Majority of their samples were run in clinical scenarios where the primary diagnosis prior to testing was defined (80%), with tubercular uveitis accounting for the most cases, 39 of 100 cases. Their study also included PCR evaluation of *P. acnes*, Eubacterium and panfungus on post-op chronic endophthalmitis cases, whereas our PCR study only evaluates viral etiologies, toxoplasmosis, and M. Tb. They found that management was changed following PCR testing in 17.7% of cases. Harper et al. published results of PCR in patients with posterior infectious uveitis, finding that management was changed in 19.5% of cases [22]. Like Kharel et al., this study was composed mostly of cases where the clinical diagnosis prior to PCR testing was well-defined and PCR testing was confirmatory in nature, with the highest percentage of cases being necrotizing herpetic retinitis (34%). Our sample set differs in that majority of our cases, the pre-testing diagnosis was unknown and thus as expected the percentage of treatment changes related to PCR were higher. Also, Harper et al. includes multiple samples from the same patient in the primary analysis, whereas in our study only the initial sample is included.

Three studies have looked specifically at aqueous humor sampling the diagnosis of infectious uveitis, as this procedure is more commonly undertaken in the office setting [23–25]. Anwar et al. analyzed anterior chamber paracentesis specifically in anterior uveitis and found a relatively low rate of treatment change at 13% in this subset of patients [23]. In our subset of cases with aqueous samples, our rate of treatment change was higher at 31%. This variation could be due to the different scenarios in which sampling was undertaken. For example, if the paracentesis was being performed specifically for diagnostic purposes versus if the main purpose of the paracentesis was intraocular pressure lowering and a specimen was then sent for PCR testing. Rothova et al. looked at PCR utility from aqueous specimens in specifically posterior uveitis finding that 24% of cases required a treatment change from results of PCR, thus suggesting that even in posterior disease anterior sampling can be of clinical use [24]. Choi et al. compared the utility of PCR samples from aqueous humor to serologic sampling in diagnosis of presumed infectious uveitis showing in general higher rates of positivity in PCR samples over serology [25].

To our knowledge, this is the first study comparing the effect of PCR from both aqueous and vitreous samples in various clinical scenarios or pre-sampling diagnoses. There is no clearly defined algorithm as to when to employ PCR sampling; it depends mainly on physician preference and practice patterns within that community. Our study encompasses a wide range of ophthalmologists, both retina and uveitis specialists, from our institution. By including data from multiple specialists, the study has inherent variation of practice patterns. However, many of these subjects were seen by the same set of retina and uveitis specialists with frequent discussions as to management and thus overlaps in care team. What agents to test for is decided by the ophthalmologist upon submitting the specimen to pathology. There is no set panel. As evidenced by our study, sampling is being undertaken most frequently when there is suspicion for herpetic etiology. Our experience shows a tendency to order PCR testing in clinical scenarios where the diagnosis is uncertain, and the anterior chamber or vitreous sampling is being undertaken for diagnostic purposes. In those cases where PCR is sent and the pre-testing diagnosis is not related to infectious agents tested for by PCR, there were no cases of diagnosis or treatment change. These patients were commonly undergoing vitreous or aqueous sampling for other reasons, like vitrectomy or paracentesis to lower intraocular pressure and PCR was then sent on the sample. Further analysis of cost into sending these samples is warranted to determine the burden on the healthcare system for these additional tests when the pre-testing utility of sampling is low.

As expected, PCR sampling had a more significant impact on diagnosis when pre-testing diagnosis was unknown, however rates of treatment change were similar between those patients where pre-testing diagnosis was unknown and those patients where sampling was confirmatory in nature (i.e. cases of Acute Retinal Necrosis). Examples of these changes in those where pre-testing diagnosis was presumed related to PCR include increases in dosage of anti-viral medication or addition of intraocular antivirals. Thus suggesting, that even when confirmatory in nature, PCR testing maintains a significant impact on patient management. These patterns persisted when subdividing specimens into those from aqueous and vitreous.

The two main limitations of this study are its small sample size and its retrospective nature. However, given it represents sampling over a 5-year period at a large tertiary care center and across multiple retina and uveitis specialists, we feel it likely gives a fair representation of PCR sampling rates and the various clinical scenarios it is undertaken in at our institution. Further prospective analyses are needed to evaluate further the impact of PCR on patient care as well as the its cost-benefit in various clinical scenarios.

## Conclusions

PCR testing is a well-accepted adjunct in the diagnosis of infectious uveitides. There is no well accepted practice pattern as to when to employ PCR testing. Our results show PCR had the highest impact on diagnosis in cases where the pre-sampling diagnosis was broad and uncertain, however even in cases where PCR was confirmatory in nature, it did still prove useful in regards to tailoring management. In those cases where pre-testing diagnosis was not related to infectious agents related to PCR, sampling did not have an impact on diagnosis or management, thus suggesting minimal utility and undue costs in PCR testing in these clinical scenarios. Larger prospective trials are needed to further delineate how PCR results from vitreous and aqueous affect patient care, as well as a cost-benefit analysis of PCR sampling.

## Data Availability

The datasets used and/or analyzed during the current study are available from the corresponding author on reasonable request.

## Abbreviations

PCR: Polymerase Chain Reaction
HSV: Herpes Simplex Virus
VZV: Varicella Zoster Virus
CMV: Cytomegalovirus
Toxoplasmosis: *Toxoplasmosis gondii*
M. tb: *Mycobacterium tuberculosis*
EBV: Epstein-Barr Virus
